# Supplementary material for: Transcriptome Analysis Reveals Vimentin-Induced Disruption of Cell–Cell Associations Augments Breast Cancer Cell Migration
Source: Cells. 2022 Dec 13;11(24):4035. doi: 10.3390/cells11244035 (PMC9776984; doi:10.3390/cells11244035)
Supplement: Supplementary file 1 [file cells-11-04035-s001.zip › DEGs and enrichment analysis.zip!/DEGs and enrichment analysis/WT_VIMvsVector KEGG/GSEA/WT_VIM/gsea_report_for_WT_VIM_1625876782417.html]

Report for WT\_VIM 1625876782417 [GSEA]


| GS   follow link to MSigDB | GS DETAILS | SIZE | ES | NES | NOM p-val | FDR q-val | FWER p-val | RANK AT MAX | LEADING EDGE || 1 | MICRORNAS\_IN\_CANCER(HSA05206) | Details ... | 245 | 0.84 | 1.40 | 0.000 | 0.165 | 0.000 | 2498 | tags=4%, list=4%, signal=4% |
| 2 | EPSTEIN\_BARR\_VIRUS\_INFECTION(HSA05169) | Details ... | 470 | 0.84 | 1.40 | 0.000 | 0.165 | 0.165 | 2636 | tags=4%, list=4%, signal=4% |
| 3 | DNA\_REPLICATION(HSA03030) | Details ... | 65 | 0.74 | 1.28 | 0.000 | 0.165 | 0.165 | 9747 | tags=49%, list=16%, signal=59% |
| 4 | STEROID\_BIOSYNTHESIS(HSA00100) | Details ... | 34 | 0.74 | 1.24 | 0.000 | 0.250 | 0.335 | 12529 | tags=53%, list=21%, signal=67% |
| 5 | BASE\_EXCISION\_REPAIR(HSA03410) | Details ... | 97 | 0.70 | 1.24 | 0.000 | 0.267 | 0.335 | 11308 | tags=38%, list=19%, signal=47% |
| 6 | RNA\_POLYMERASE(HSA03020) | Details ... | 54 | 0.68 | 1.20 | 0.000 | 0.250 | 0.335 | 17258 | tags=61%, list=29%, signal=86% |
| 7 | RIBOSOME(HSA03010) | Details ... | 2361 | 0.65 | 1.20 | 0.000 | 0.238 | 0.335 | 15878 | tags=23%, list=27%, signal=30% |
| 8 | TASTE\_TRANSDUCTION(HSA04742) | Details ... | 107 | 0.70 | 1.19 | 0.000 | 0.249 | 0.498 | 11398 | tags=26%, list=19%, signal=32% |
| 9 | MATURITY\_ONSET\_DIABETES\_OF\_THE\_YOUNG(HSA04950) | Details ... | 26 | 0.72 | 1.19 | 0.000 | 0.240 | 0.498 | 10619 | tags=46%, list=18%, signal=56% |
| 10 | SYSTEMIC\_LUPUS\_ERYTHEMATOSUS(HSA05322) | Details ... | 290 | 0.66 | 1.18 | 0.000 | 0.232 | 0.498 | 13489 | tags=31%, list=23%, signal=40% |
| 11 | BIOSYNTHESIS\_OF\_AMINO\_ACIDS(HSA01230) | Details ... | 258 | 0.68 | 1.17 | 0.000 | 0.241 | 0.498 | 11800 | tags=29%, list=20%, signal=36% |
| 12 | 2\_OXOCARBOXYLIC\_ACID\_METABOLISM(HSA01210) | Details ... | 37 | 0.70 | 1.16 | 0.000 | 0.304 | 0.498 | 11800 | tags=49%, list=20%, signal=61% |
| 13 | MISMATCH\_REPAIR(HSA03430) | Details ... | 59 | 0.67 | 1.16 | 0.000 | 0.293 | 0.498 | 17101 | tags=56%, list=29%, signal=79% |
| 14 | CITRATE\_CYCLE\_(TCA\_CYCLE)(HSA00020) | Details ... | 70 | 0.66 | 1.16 | 0.000 | 0.284 | 0.498 | 16530 | tags=50%, list=28%, signal=69% |
| 15 | SPLICEOSOME(HSA03040) | Details ... | 457 | 0.64 | 1.16 | 0.000 | 0.287 | 0.498 | 16935 | tags=40%, list=29%, signal=56% |
| 16 | GLYCOSPHINGOLIPID\_BIOSYNTHESIS\_\_\_GLOBO\_AND\_ISOGLOBO\_SERIES(HSA00603) | Details ... | 21 | 0.67 | 1.15 | 0.000 | 0.290 | 0.498 | 9884 | tags=52%, list=17%, signal=63% |
| 17 | NEUROACTIVE\_LIGAND\_RECEPTOR\_INTERACTION(HSA04080) | Details ... | 405 | 0.66 | 1.15 | 0.000 | 0.293 | 0.498 | 10324 | tags=31%, list=17%, signal=38% |
| 18 | PHOTOTRANSDUCTION(HSA04744) | Details ... | 42 | 0.66 | 1.15 | 0.000 | 0.285 | 0.498 | 15698 | tags=43%, list=27%, signal=58% |
| 19 | CHOLESTEROL\_METABOLISM(HSA04979) | Details ... | 81 | 0.67 | 1.15 | 0.000 | 0.288 | 0.498 | 12421 | tags=27%, list=21%, signal=34% |
| 20 | ARGININE\_AND\_PROLINE\_METABOLISM(HSA00330) | Details ... | 114 | 0.66 | 1.14 | 0.000 | 0.289 | 0.652 | 11663 | tags=31%, list=20%, signal=38% |
| 21 | TAURINE\_AND\_HYPOTAURINE\_METABOLISM(HSA00430) |  | 20 | 0.70 | 1.14 | 0.000 | 0.283 | 0.652 | 3605 | tags=20%, list=6%, signal=21% |
| 22 | GLUTATHIONE\_METABOLISM(HSA00480) |  | 112 | 0.65 | 1.14 | 0.000 | 0.278 | 0.652 | 11380 | tags=31%, list=19%, signal=39% |
| 23 | PENTOSE\_PHOSPHATE\_PATHWAY(HSA00030) |  | 54 | 0.68 | 1.14 | 0.170 | 0.273 | 0.652 | 11258 | tags=44%, list=19%, signal=55% |
| 24 | PROTEASOME(HSA03050) |  | 116 | 0.65 | 1.14 | 0.000 | 0.269 | 0.652 | 15433 | tags=47%, list=26%, signal=63% |
| 25 | ESTROGEN\_SIGNALING\_PATHWAY(HSA04915) |  | 330 | 0.64 | 1.14 | 0.000 | 0.264 | 0.652 | 16065 | tags=38%, list=27%, signal=53% |
| 26 | CARBOHYDRATE\_DIGESTION\_AND\_ABSORPTION(HSA04973) |  | 66 | 0.65 | 1.14 | 0.000 | 0.261 | 0.652 | 11211 | tags=30%, list=19%, signal=37% |
| 27 | GLYOXYLATE\_AND\_DICARBOXYLATE\_METABOLISM(HSA00630) |  | 66 | 0.65 | 1.14 | 0.000 | 0.269 | 0.652 | 17550 | tags=48%, list=30%, signal=69% |
| 28 | ALCOHOLISM(HSA05034) |  | 303 | 0.64 | 1.13 | 0.000 | 0.271 | 0.652 | 16882 | tags=51%, list=29%, signal=72% |
| 29 | GNRH\_SECRETION(HSA04929) |  | 87 | 0.65 | 1.13 | 0.000 | 0.273 | 0.652 | 16280 | tags=55%, list=28%, signal=76% |
| 30 | PRION\_DISEASES(HSA05020) |  | 56 | 0.64 | 1.13 | 0.000 | 0.269 | 0.652 | 16559 | tags=52%, list=28%, signal=72% |
| 31 | FRUCTOSE\_AND\_MANNOSE\_METABOLISM(HSA00051) |  | 62 | 0.68 | 1.13 | 0.000 | 0.272 | 0.652 | 9785 | tags=42%, list=17%, signal=50% |
| 32 | GLUTAMATERGIC\_SYNAPSE(HSA04724) |  | 152 | 0.64 | 1.13 | 0.000 | 0.274 | 0.652 | 17148 | tags=51%, list=29%, signal=72% |
| 33 | PYRIMIDINE\_METABOLISM(HSA00240) |  | 104 | 0.64 | 1.13 | 0.000 | 0.270 | 0.652 | 13116 | tags=43%, list=22%, signal=56% |
| 34 | BIOSYNTHESIS\_OF\_UNSATURATED\_FATTY\_ACIDS(HSA01040) |  | 41 | 0.64 | 1.13 | 0.000 | 0.267 | 0.652 | 17670 | tags=63%, list=30%, signal=90% |
| 35 | ONE\_CARBON\_POOL\_BY\_FOLATE(HSA00670) |  | 53 | 0.64 | 1.13 | 0.000 | 0.264 | 0.652 | 19778 | tags=47%, list=33%, signal=71% |
| 36 | TYROSINE\_METABOLISM(HSA00350) |  | 70 | 0.68 | 1.13 | 0.000 | 0.262 | 0.652 | 11555 | tags=33%, list=20%, signal=41% |
| 37 | STAPHYLOCOCCUS\_AUREUS\_INFECTION(HSA05150) |  | 288 | 0.65 | 1.13 | 0.170 | 0.259 | 0.652 | 11426 | tags=17%, list=19%, signal=21% |
| 38 | HOMOLOGOUS\_RECOMBINATION(HSA03440) |  | 69 | 0.66 | 1.13 | 0.000 | 0.261 | 0.652 | 16461 | tags=59%, list=28%, signal=82% |
| 39 | CELL\_CYCLE(HSA04110) |  | 267 | 0.62 | 1.13 | 0.000 | 0.258 | 0.652 | 18450 | tags=51%, list=31%, signal=74% |
| 40 | GASTRIC\_ACID\_SECRETION(HSA04971) |  | 142 | 0.64 | 1.12 | 0.000 | 0.264 | 0.652 | 12565 | tags=37%, list=21%, signal=46% |
| 41 | RNA\_TRANSPORT(HSA03013) |  | 443 | 0.63 | 1.12 | 0.000 | 0.261 | 0.652 | 18587 | tags=47%, list=31%, signal=69% |
| 42 | ARGININE\_BIOSYNTHESIS(HSA00220) |  | 71 | 0.65 | 1.12 | 0.000 | 0.278 | 0.652 | 16308 | tags=31%, list=28%, signal=43% |
| 43 | CYSTEINE\_AND\_METHIONINE\_METABOLISM(HSA00270) |  | 116 | 0.63 | 1.12 | 0.000 | 0.279 | 0.652 | 18974 | tags=44%, list=32%, signal=65% |
| 44 | ANTIGEN\_PROCESSING\_AND\_PRESENTATION(HSA04612) |  | 197 | 0.64 | 1.11 | 0.170 | 0.307 | 0.652 | 14100 | tags=28%, list=24%, signal=37% |
| 45 | RETROGRADE\_ENDOCANNABINOID\_SIGNALING(HSA04723) |  | 498 | 0.60 | 1.11 | 0.000 | 0.304 | 0.652 | 17562 | tags=24%, list=30%, signal=33% |
| 46 | THYROID\_CANCER(HSA05216) |  | 54 | 0.64 | 1.11 | 0.000 | 0.308 | 0.652 | 21104 | tags=80%, list=36%, signal=124% |
| 47 | ANTIFOLATE\_RESISTANCE(HSA01523) |  | 52 | 0.63 | 1.11 | 0.000 | 0.319 | 0.652 | 19381 | tags=54%, list=33%, signal=80% |
| 48 | CARBON\_METABOLISM(HSA01200) |  | 342 | 0.62 | 1.11 | 0.000 | 0.315 | 0.652 | 17761 | tags=40%, list=30%, signal=57% |
| 49 | OVARIAN\_STEROIDOGENESIS(HSA04913) |  | 77 | 0.65 | 1.11 | 0.000 | 0.312 | 0.652 | 14096 | tags=43%, list=24%, signal=56% |
| 50 | N\_GLYCAN\_BIOSYNTHESIS(HSA00510) |  | 79 | 0.62 | 1.10 | 0.000 | 0.319 | 0.652 | 17468 | tags=58%, list=30%, signal=83% |
| 51 | RIBOSOME\_BIOGENESIS\_IN\_EUKARYOTES(HSA03008) |  | 202 | 0.62 | 1.10 | 0.000 | 0.320 | 0.652 | 19604 | tags=49%, list=33%, signal=73% |
| 52 | GAP\_JUNCTION(HSA04540) |  | 165 | 0.62 | 1.10 | 0.000 | 0.323 | 0.652 | 16477 | tags=42%, list=28%, signal=58% |
| 53 | GABAERGIC\_SYNAPSE(HSA04727) |  | 134 | 0.62 | 1.10 | 0.000 | 0.320 | 0.652 | 12565 | tags=34%, list=21%, signal=43% |
| 54 | BLADDER\_CANCER(HSA05219) |  | 69 | 0.63 | 1.10 | 0.000 | 0.317 | 0.652 | 19512 | tags=58%, list=33%, signal=86% |
| 55 | PARKINSON\_DISEASE(HSA05012) |  | 1234 | 0.59 | 1.10 | 0.000 | 0.314 | 0.652 | 18078 | tags=26%, list=31%, signal=37% |
| 56 | CHOLINERGIC\_SYNAPSE(HSA04725) |  | 151 | 0.61 | 1.09 | 0.000 | 0.320 | 0.652 | 16065 | tags=43%, list=27%, signal=59% |
| 57 | OXIDATIVE\_PHOSPHORYLATION(HSA00190) |  | 871 | 0.58 | 1.09 | 0.000 | 0.326 | 0.652 | 19262 | tags=22%, list=33%, signal=32% |
| 58 | GLYCOSYLPHOSPHATIDYLINOSITOL\_(GPI)\_ANCHOR\_BIOSYNTHESIS(HSA00563) |  | 46 | 0.62 | 1.09 | 0.000 | 0.324 | 0.652 | 21512 | tags=67%, list=36%, signal=106% |
| 59 | PHENYLALANINE\_METABOLISM(HSA00360) |  | 36 | 0.67 | 1.09 | 0.170 | 0.321 | 0.652 | 11253 | tags=31%, list=19%, signal=38% |
| 60 | CYTOSOLIC\_DNA\_SENSING\_PATHWAY(HSA04623) |  | 91 | 0.62 | 1.09 | 0.000 | 0.321 | 0.652 | 19386 | tags=44%, list=33%, signal=65% |
| 61 | MINERAL\_ABSORPTION(HSA04978) |  | 129 | 0.62 | 1.09 | 0.000 | 0.321 | 0.652 | 15529 | tags=36%, list=26%, signal=49% |
| 62 | PROGESTERONE\_MEDIATED\_OOCYTE\_MATURATION(HSA04914) |  | 182 | 0.60 | 1.09 | 0.000 | 0.319 | 0.652 | 18956 | tags=53%, list=32%, signal=78% |
| 63 | MELANOGENESIS(HSA04916) |  | 132 | 0.62 | 1.09 | 0.000 | 0.316 | 0.652 | 17677 | tags=58%, list=30%, signal=82% |
| 64 | FANCONI\_ANEMIA\_PATHWAY(HSA03460) |  | 91 | 0.62 | 1.09 | 0.000 | 0.314 | 0.652 | 17706 | tags=65%, list=30%, signal=92% |
| 65 | PYRUVATE\_METABOLISM(HSA00620) |  | 70 | 0.62 | 1.09 | 0.000 | 0.312 | 0.652 | 17340 | tags=50%, list=29%, signal=71% |
| 66 | NUCLEOTIDE\_EXCISION\_REPAIR(HSA03420) |  | 83 | 0.63 | 1.09 | 0.000 | 0.314 | 0.652 | 21243 | tags=64%, list=36%, signal=100% |
| 67 | FOLATE\_BIOSYNTHESIS(HSA00790) |  | 53 | 0.62 | 1.09 | 0.000 | 0.312 | 0.652 | 18886 | tags=45%, list=32%, signal=66% |
| 68 | ALZHEIMER\_DISEASE(HSA05010) |  | 1550 | 0.59 | 1.09 | 0.000 | 0.310 | 0.652 | 19339 | tags=30%, list=33%, signal=44% |
| 69 | MRNA\_SURVEILLANCE\_PATHWAY(HSA03015) |  | 189 | 0.62 | 1.09 | 0.000 | 0.308 | 0.652 | 20325 | tags=61%, list=34%, signal=93% |
| 70 | CENTRAL\_CARBON\_METABOLISM\_IN\_CANCER(HSA05230) |  | 146 | 0.61 | 1.09 | 0.000 | 0.310 | 0.652 | 17371 | tags=47%, list=29%, signal=67% |
| 71 | OOCYTE\_MEIOSIS(HSA04114) |  | 258 | 0.60 | 1.09 | 0.000 | 0.313 | 0.817 | 20204 | tags=57%, list=34%, signal=86% |
| 72 | ABC\_TRANSPORTERS(HSA02010) |  | 75 | 0.64 | 1.09 | 0.000 | 0.311 | 0.817 | 10636 | tags=25%, list=18%, signal=31% |
| 73 | GLYCOLYSIS\_\_\_GLUCONEOGENESIS(HSA00010) |  | 217 | 0.61 | 1.08 | 0.170 | 0.311 | 0.817 | 15982 | tags=32%, list=27%, signal=43% |
| 74 | MANNOSE\_TYPE\_O\_GLYCAN\_BIOSYNTHESIS(HSA00515) |  | 27 | 0.64 | 1.08 | 0.170 | 0.318 | 1.000 | 19016 | tags=78%, list=32%, signal=115% |
| 75 | NICOTINE\_ADDICTION(HSA05033) |  | 43 | 0.64 | 1.08 | 0.170 | 0.321 | 1.000 | 17148 | tags=28%, list=29%, signal=39% |
| 76 | THYROID\_HORMONE\_SIGNALING\_PATHWAY(HSA04919) |  | 225 | 0.60 | 1.08 | 0.000 | 0.325 | 1.000 | 16892 | tags=47%, list=29%, signal=65% |
| 77 | AMINOACYL\_TRNA\_BIOSYNTHESIS(HSA00970) |  | 72 | 0.63 | 1.08 | 0.000 | 0.327 | 1.000 | 19261 | tags=65%, list=33%, signal=97% |
| 78 | GLYCOSPHINGOLIPID\_BIOSYNTHESIS\_\_\_LACTO\_AND\_NEOLACTO\_SERIES(HSA00601) |  | 40 | 0.61 | 1.08 | 0.000 | 0.325 | 1.000 | 13283 | tags=40%, list=22%, signal=52% |
| 79 | PORPHYRIN\_AND\_CHLOROPHYLL\_METABOLISM(HSA00860) |  | 103 | 0.61 | 1.08 | 0.000 | 0.323 | 1.000 | 17268 | tags=33%, list=29%, signal=47% |
| 80 | LONG\_TERM\_DEPRESSION(HSA04730) |  | 81 | 0.62 | 1.08 | 0.000 | 0.323 | 1.000 | 18247 | tags=56%, list=31%, signal=80% |
| 81 | HUNTINGTON\_DISEASE(HSA05016) |  | 1403 | 0.58 | 1.08 | 0.000 | 0.333 | 1.000 | 19400 | tags=28%, list=33%, signal=41% |
| 82 | BUTANOATE\_METABOLISM(HSA00650) |  | 46 | 0.63 | 1.08 | 0.000 | 0.331 | 1.000 | 17627 | tags=54%, list=30%, signal=77% |
| 83 | NITROGEN\_METABOLISM(HSA00910) |  | 35 | 0.64 | 1.08 | 0.170 | 0.329 | 1.000 | 16834 | tags=43%, list=28%, signal=60% |
| 84 | INSULIN\_SECRETION(HSA04911) |  | 110 | 0.62 | 1.08 | 0.154 | 0.327 | 1.000 | 16065 | tags=47%, list=27%, signal=65% |
| 85 | TERPENOID\_BACKBONE\_BIOSYNTHESIS(HSA00900) |  | 50 | 0.61 | 1.08 | 0.000 | 0.325 | 1.000 | 20515 | tags=56%, list=35%, signal=86% |
| 86 | ENDOCRINE\_AND\_OTHER\_FACTOR\_REGULATED\_CALCIUM\_REABSORPTION(HSA04961) |  | 89 | 0.62 | 1.07 | 0.000 | 0.329 | 1.000 | 16645 | tags=44%, list=28%, signal=61% |
| 87 | HUMAN\_T\_CELL\_LEUKEMIA\_VIRUS\_1\_INFECTION(HSA05166) |  | 435 | 0.59 | 1.07 | 0.000 | 0.327 | 1.000 | 19523 | tags=47%, list=33%, signal=70% |
| 88 | DRUG\_METABOLISM\_\_\_OTHER\_ENZYMES(HSA00983) |  | 210 | 0.59 | 1.07 | 0.000 | 0.325 | 1.000 | 14467 | tags=29%, list=24%, signal=38% |
| 89 | PARATHYROID\_HORMONE\_SYNTHESIS\_\_SECRETION\_AND\_ACTION(HSA04928) |  | 156 | 0.62 | 1.07 | 0.000 | 0.325 | 1.000 | 16685 | tags=49%, list=28%, signal=69% |
| 90 | PRIMARY\_IMMUNODEFICIENCY(HSA05340) |  | 131 | 0.67 | 1.07 | 0.000 | 0.324 | 1.000 | 13038 | tags=19%, list=22%, signal=24% |
| 91 | BREAST\_CANCER(HSA05224) |  | 212 | 0.60 | 1.07 | 0.000 | 0.322 | 1.000 | 20328 | tags=59%, list=34%, signal=90% |
| 92 | NECROPTOSIS(HSA04217) |  | 455 | 0.59 | 1.07 | 0.000 | 0.320 | 1.000 | 15554 | tags=32%, list=26%, signal=43% |
| 93 | VIRAL\_CARCINOGENESIS(HSA05203) |  | 437 | 0.60 | 1.07 | 0.000 | 0.319 | 1.000 | 19512 | tags=50%, list=33%, signal=74% |
| 94 | INTESTINAL\_IMMUNE\_NETWORK\_FOR\_IGA\_PRODUCTION(HSA04672) |  | 127 | 0.61 | 1.07 | 0.154 | 0.317 | 1.000 | 12572 | tags=13%, list=21%, signal=16% |
| 95 | ASTHMA(HSA05310) |  | 100 | 0.55 | 1.07 | 0.000 | 0.321 | 1.000 | 10539 | tags=6%, list=18%, signal=7% |
| 96 | RNA\_DEGRADATION(HSA03018) |  | 171 | 0.60 | 1.07 | 0.000 | 0.319 | 1.000 | 20601 | tags=56%, list=35%, signal=85% |
| 97 | AMINO\_SUGAR\_AND\_NUCLEOTIDE\_SUGAR\_METABOLISM(HSA00520) |  | 69 | 0.60 | 1.07 | 0.000 | 0.318 | 1.000 | 22743 | tags=75%, list=38%, signal=122% |
| 98 | CALCIUM\_SIGNALING\_PATHWAY(HSA04020) |  | 371 | 0.60 | 1.07 | 0.000 | 0.318 | 1.000 | 16241 | tags=35%, list=27%, signal=48% |
| 99 | APOPTOSIS(HSA04210) |  | 302 | 0.60 | 1.07 | 0.170 | 0.321 | 1.000 | 20359 | tags=49%, list=34%, signal=75% |
| 100 | P53\_SIGNALING\_PATHWAY(HSA04115) |  | 164 | 0.59 | 1.07 | 0.000 | 0.320 | 1.000 | 20359 | tags=43%, list=34%, signal=66% |
| 101 | GALACTOSE\_METABOLISM(HSA00052) |  | 50 | 0.62 | 1.07 | 0.170 | 0.318 | 1.000 | 8288 | tags=28%, list=14%, signal=33% |
| 102 | VASCULAR\_SMOOTH\_MUSCLE\_CONTRACTION(HSA04270) |  | 216 | 0.61 | 1.07 | 0.000 | 0.317 | 1.000 | 18447 | tags=45%, list=31%, signal=65% |
| 103 | HUMAN\_IMMUNODEFICIENCY\_VIRUS\_1\_INFECTION(HSA05170) |  | 445 | 0.59 | 1.07 | 0.000 | 0.317 | 1.000 | 19603 | tags=42%, list=33%, signal=63% |
| 104 | PROPANOATE\_METABOLISM(HSA00640) |  | 62 | 0.61 | 1.07 | 0.000 | 0.321 | 1.000 | 21511 | tags=63%, list=36%, signal=99% |
| 105 | PENTOSE\_AND\_GLUCURONATE\_INTERCONVERSIONS(HSA00040) |  | 106 | 0.59 | 1.07 | 0.000 | 0.330 | 1.000 | 18385 | tags=27%, list=31%, signal=40% |
| 106 | FATTY\_ACID\_BIOSYNTHESIS(HSA00061) |  | 27 | 0.61 | 1.07 | 0.000 | 0.328 | 1.000 | 22643 | tags=74%, list=38%, signal=120% |
| 107 | PROXIMAL\_TUBULE\_BICARBONATE\_RECLAMATION(HSA04964) |  | 42 | 0.61 | 1.07 | 0.000 | 0.328 | 1.000 | 16834 | tags=48%, list=28%, signal=67% |
| 108 | THERMOGENESIS(HSA04714) |  | 1057 | 0.57 | 1.06 | 0.000 | 0.334 | 1.000 | 22738 | tags=30%, list=38%, signal=47% |
| 109 | LONGEVITY\_REGULATING\_PATHWAY\_\_\_MULTIPLE\_SPECIES(HSA04213) |  | 121 | 0.59 | 1.06 | 0.000 | 0.334 | 1.000 | 16306 | tags=40%, list=28%, signal=55% |
| 110 | VEGF\_SIGNALING\_PATHWAY(HSA04370) |  | 90 | 0.60 | 1.06 | 0.000 | 0.339 | 1.000 | 19512 | tags=60%, list=33%, signal=89% |
| 111 | RAP1\_SIGNALING\_PATHWAY(HSA04015) |  | 364 | 0.60 | 1.06 | 0.000 | 0.339 | 1.000 | 21106 | tags=55%, list=36%, signal=85% |
| 112 | MORPHINE\_ADDICTION(HSA05032) |  | 118 | 0.60 | 1.06 | 0.000 | 0.337 | 1.000 | 17148 | tags=37%, list=29%, signal=52% |
| 113 | THYROID\_HORMONE\_SYNTHESIS(HSA04918) |  | 99 | 0.60 | 1.06 | 0.000 | 0.340 | 1.000 | 17160 | tags=47%, list=29%, signal=67% |
| 114 | MELANOMA(HSA05218) |  | 109 | 0.60 | 1.06 | 0.000 | 0.338 | 1.000 | 21791 | tags=58%, list=37%, signal=91% |
| 115 | HEPATOCELLULAR\_CARCINOMA(HSA05225) |  | 313 | 0.59 | 1.06 | 0.000 | 0.341 | 1.000 | 21955 | tags=58%, list=37%, signal=91% |
| 116 | UBIQUINONE\_AND\_OTHER\_TERPENOID\_QUINONE\_BIOSYNTHESIS(HSA00130) |  | 20 | 0.65 | 1.06 | 0.000 | 0.341 | 1.000 | 16745 | tags=50%, list=28%, signal=70% |
| 117 | GLYCINE\_\_SERINE\_AND\_THREONINE\_METABOLISM(HSA00260) |  | 95 | 0.60 | 1.06 | 0.170 | 0.339 | 1.000 | 12172 | tags=27%, list=21%, signal=34% |
| 118 | PURINE\_METABOLISM(HSA00230) |  | 246 | 0.58 | 1.06 | 0.000 | 0.341 | 1.000 | 18920 | tags=45%, list=32%, signal=66% |
| 119 | ENDOCRINE\_RESISTANCE(HSA01522) |  | 139 | 0.60 | 1.06 | 0.000 | 0.342 | 1.000 | 20326 | tags=58%, list=34%, signal=89% |
| 120 | PLATELET\_ACTIVATION(HSA04611) |  | 205 | 0.59 | 1.06 | 0.000 | 0.343 | 1.000 | 19826 | tags=51%, list=34%, signal=76% |
| 121 | NON\_HOMOLOGOUS\_END\_JOINING(HSA03450) |  | 25 | 0.62 | 1.06 | 0.183 | 0.351 | 1.000 | 12815 | tags=44%, list=22%, signal=56% |
| 122 | VIRAL\_MYOCARDITIS(HSA05416) |  | 267 | 0.60 | 1.06 | 0.324 | 0.350 | 1.000 | 20131 | tags=23%, list=34%, signal=34% |
| 123 | FATTY\_ACID\_ELONGATION(HSA00062) |  | 44 | 0.60 | 1.06 | 0.000 | 0.350 | 1.000 | 17670 | tags=59%, list=30%, signal=84% |
| 124 | VARIOUS\_TYPES\_OF\_N\_GLYCAN\_BIOSYNTHESIS(HSA00513) |  | 65 | 0.61 | 1.06 | 0.154 | 0.356 | 1.000 | 21578 | tags=66%, list=36%, signal=104% |
| 125 | SALMONELLA\_INFECTION(HSA05132) |  | 613 | 0.58 | 1.06 | 0.000 | 0.356 | 1.000 | 21784 | tags=45%, list=37%, signal=70% |
| 126 | BACTERIAL\_INVASION\_OF\_EPITHELIAL\_CELLS(HSA05100) |  | 198 | 0.60 | 1.06 | 0.000 | 0.354 | 1.000 | 21997 | tags=53%, list=37%, signal=84% |
| 127 | CHRONIC\_MYELOID\_LEUKEMIA(HSA05220) |  | 141 | 0.60 | 1.06 | 0.000 | 0.357 | 1.000 | 21791 | tags=62%, list=37%, signal=99% |
| 128 | SPHINGOLIPID\_SIGNALING\_PATHWAY(HSA04071) |  | 173 | 0.60 | 1.05 | 0.000 | 0.360 | 1.000 | 19799 | tags=59%, list=33%, signal=88% |
| 129 | METABOLIC\_PATHWAYS(HSA01100) |  | 3335 | 0.58 | 1.05 | 0.000 | 0.361 | 1.000 | 20344 | tags=41%, list=34%, signal=59% |
| 130 | THIAMINE\_METABOLISM(HSA00730) |  | 28 | 0.63 | 1.05 | 0.000 | 0.364 | 1.000 | 20623 | tags=64%, list=35%, signal=99% |
| 131 | NEUROTROPHIN\_SIGNALING\_PATHWAY(HSA04722) |  | 207 | 0.59 | 1.05 | 0.000 | 0.362 | 1.000 | 22747 | tags=64%, list=38%, signal=104% |
| 132 | HIPPO\_SIGNALING\_PATHWAY(HSA04390) |  | 284 | 0.59 | 1.05 | 0.324 | 0.367 | 1.000 | 21983 | tags=60%, list=37%, signal=94% |
| 133 | CELLULAR\_SENESCENCE(HSA04218) |  | 309 | 0.59 | 1.05 | 0.000 | 0.369 | 1.000 | 19621 | tags=50%, list=33%, signal=74% |
| 134 | ALDOSTERONE\_REGULATED\_SODIUM\_REABSORPTION(HSA04960) |  | 49 | 0.59 | 1.05 | 0.000 | 0.368 | 1.000 | 23896 | tags=73%, list=40%, signal=123% |
| 135 | ASCORBATE\_AND\_ALDARATE\_METABOLISM(HSA00053) |  | 74 | 0.59 | 1.05 | 0.183 | 0.366 | 1.000 | 12482 | tags=23%, list=21%, signal=29% |
| 136 | VASOPRESSIN\_REGULATED\_WATER\_REABSORPTION(HSA04962) |  | 68 | 0.59 | 1.05 | 0.000 | 0.365 | 1.000 | 22097 | tags=62%, list=37%, signal=98% |
| 137 | GASTRIC\_CANCER(HSA05226) |  | 211 | 0.59 | 1.05 | 0.170 | 0.364 | 1.000 | 21955 | tags=61%, list=37%, signal=96% |
| 138 | CIRCADIAN\_ENTRAINMENT(HSA04713) |  | 134 | 0.60 | 1.05 | 0.000 | 0.364 | 1.000 | 18016 | tags=49%, list=30%, signal=71% |
| 139 | PHAGOSOME(HSA04145) |  | 431 | 0.58 | 1.05 | 0.170 | 0.370 | 1.000 | 20357 | tags=34%, list=34%, signal=52% |
| 140 | RENIN\_ANGIOTENSIN\_SYSTEM(HSA04614) |  | 32 | 0.60 | 1.05 | 0.000 | 0.370 | 1.000 | 16543 | tags=38%, list=28%, signal=52% |
| 141 | NON\_SMALL\_CELL\_LUNG\_CANCER(HSA05223) |  | 96 | 0.60 | 1.05 | 0.000 | 0.368 | 1.000 | 17982 | tags=58%, list=30%, signal=84% |
| 142 | NON\_ALCOHOLIC\_FATTY\_LIVER\_DISEASE\_(NAFLD)(HSA04932) |  | 631 | 0.57 | 1.05 | 0.000 | 0.370 | 1.000 | 18979 | tags=27%, list=32%, signal=39% |
| 143 | SALIVARY\_SECRETION(HSA04970) |  | 116 | 0.59 | 1.05 | 0.000 | 0.370 | 1.000 | 15945 | tags=42%, list=27%, signal=58% |
| 144 | KAPOSI\_SARCOMA\_ASSOCIATED\_HERPESVIRUS\_INFECTION(HSA05167) |  | 392 | 0.58 | 1.05 | 0.000 | 0.373 | 1.000 | 19512 | tags=41%, list=33%, signal=61% |
| 145 | REGULATION\_OF\_ACTIN\_CYTOSKELETON(HSA04810) |  | 424 | 0.58 | 1.05 | 0.170 | 0.372 | 1.000 | 22611 | tags=54%, list=38%, signal=87% |
| 146 | RENAL\_CELL\_CARCINOMA(HSA05211) |  | 152 | 0.59 | 1.05 | 0.000 | 0.373 | 1.000 | 22747 | tags=52%, list=38%, signal=84% |
| 147 | PATHOGENIC\_ESCHERICHIA\_COLI\_INFECTION(HSA05130) |  | 586 | 0.58 | 1.05 | 0.000 | 0.375 | 1.000 | 21590 | tags=38%, list=37%, signal=60% |
| 148 | SIGNALING\_PATHWAYS\_REGULATING\_PLURIPOTENCY\_OF\_STEM\_CELLS(HSA04550) |  | 205 | 0.59 | 1.05 | 0.000 | 0.375 | 1.000 | 22747 | tags=63%, list=38%, signal=102% |
| 149 | VIBRIO\_CHOLERAE\_INFECTION(HSA05110) |  | 131 | 0.58 | 1.05 | 0.170 | 0.376 | 1.000 | 22594 | tags=52%, list=38%, signal=84% |
| 150 | FC\_GAMMA\_R\_MEDIATED\_PHAGOCYTOSIS(HSA04666) |  | 229 | 0.59 | 1.05 | 0.000 | 0.375 | 1.000 | 21751 | tags=42%, list=37%, signal=66% |
| 151 | LEGIONELLOSIS(HSA05134) |  | 290 | 0.58 | 1.05 | 0.000 | 0.377 | 1.000 | 19823 | tags=30%, list=34%, signal=44% |
| 152 | BASAL\_CELL\_CARCINOMA(HSA05217) |  | 82 | 0.61 | 1.05 | 0.324 | 0.375 | 1.000 | 15176 | tags=56%, list=26%, signal=75% |
| 153 | HUMAN\_CYTOMEGALOVIRUS\_INFECTION(HSA05163) |  | 415 | 0.58 | 1.05 | 0.000 | 0.377 | 1.000 | 19350 | tags=44%, list=33%, signal=64% |
| 154 | RELAXIN\_SIGNALING\_PATHWAY(HSA04926) |  | 183 | 0.59 | 1.05 | 0.000 | 0.381 | 1.000 | 16065 | tags=41%, list=27%, signal=56% |
| 155 | LEISHMANIASIS(HSA05140) |  | 230 | 0.57 | 1.04 | 0.154 | 0.391 | 1.000 | 15645 | tags=20%, list=26%, signal=26% |
| 156 | PROLACTIN\_SIGNALING\_PATHWAY(HSA04917) |  | 99 | 0.59 | 1.04 | 0.000 | 0.394 | 1.000 | 23256 | tags=69%, list=39%, signal=113% |
| 157 | GLIOMA(HSA05214) |  | 115 | 0.59 | 1.04 | 0.000 | 0.398 | 1.000 | 20328 | tags=61%, list=34%, signal=93% |
| 158 | GLYCOSAMINOGLYCAN\_DEGRADATION(HSA00531) |  | 38 | 0.60 | 1.04 | 0.170 | 0.398 | 1.000 | 20004 | tags=66%, list=34%, signal=99% |
| 159 | HEDGEHOG\_SIGNALING\_PATHWAY(HSA04340) |  | 83 | 0.59 | 1.04 | 0.324 | 0.402 | 1.000 | 20247 | tags=54%, list=34%, signal=82% |
| 160 | SHIGELLOSIS(HSA05131) |  | 693 | 0.58 | 1.04 | 0.000 | 0.401 | 1.000 | 23000 | tags=48%, list=39%, signal=77% |
| 161 | CUSHING\_SYNDROME(HSA04934) |  | 231 | 0.58 | 1.04 | 0.000 | 0.403 | 1.000 | 22252 | tags=59%, list=38%, signal=94% |
| 162 | NOTCH\_SIGNALING\_PATHWAY(HSA04330) |  | 91 | 0.60 | 1.04 | 0.154 | 0.405 | 1.000 | 20267 | tags=57%, list=34%, signal=87% |
| 163 | PROTEIN\_PROCESSING\_IN\_ENDOPLASMIC\_RETICULUM(HSA04141) |  | 326 | 0.59 | 1.04 | 0.000 | 0.407 | 1.000 | 23102 | tags=60%, list=39%, signal=99% |
| 164 | INFLAMMATORY\_MEDIATOR\_REGULATION\_OF\_TRP\_CHANNELS(HSA04750) |  | 134 | 0.59 | 1.04 | 0.000 | 0.407 | 1.000 | 19478 | tags=54%, list=33%, signal=81% |
| 165 | WNT\_SIGNALING\_PATHWAY(HSA04310) |  | 246 | 0.59 | 1.04 | 0.154 | 0.409 | 1.000 | 22299 | tags=60%, list=38%, signal=96% |
| 166 | MTOR\_SIGNALING\_PATHWAY(HSA04150) |  | 275 | 0.58 | 1.04 | 0.324 | 0.413 | 1.000 | 23896 | tags=68%, list=40%, signal=114% |
| 167 | PEROXISOME(HSA04146) |  | 152 | 0.58 | 1.04 | 0.170 | 0.417 | 1.000 | 23144 | tags=53%, list=39%, signal=87% |
| 168 | PD\_L1\_EXPRESSION\_AND\_PD\_1\_CHECKPOINT\_PATHWAY\_IN\_CANCER(HSA05235) |  | 133 | 0.59 | 1.04 | 0.000 | 0.417 | 1.000 | 20351 | tags=62%, list=34%, signal=94% |
| 169 | BASAL\_TRANSCRIPTION\_FACTORS(HSA03022) |  | 118 | 0.59 | 1.04 | 0.165 | 0.418 | 1.000 | 22786 | tags=53%, list=39%, signal=85% |
| 170 | AFRICAN\_TRYPANOSOMIASIS(HSA05143) |  | 107 | 0.59 | 1.04 | 0.333 | 0.417 | 1.000 | 15945 | tags=15%, list=27%, signal=20% |
| 171 | UBIQUITIN\_MEDIATED\_PROTEOLYSIS(HSA04120) |  | 384 | 0.58 | 1.04 | 0.000 | 0.415 | 1.000 | 22349 | tags=49%, list=38%, signal=79% |
| 172 | ENDOMETRIAL\_CANCER(HSA05213) |  | 96 | 0.59 | 1.04 | 0.000 | 0.415 | 1.000 | 22789 | tags=68%, list=39%, signal=110% |
| 173 | B\_CELL\_RECEPTOR\_SIGNALING\_PATHWAY(HSA04662) |  | 180 | 0.58 | 1.04 | 0.000 | 0.414 | 1.000 | 22747 | tags=38%, list=38%, signal=61% |
| 174 | LYSINE\_DEGRADATION(HSA00310) |  | 112 | 0.59 | 1.04 | 0.000 | 0.412 | 1.000 | 21515 | tags=67%, list=36%, signal=105% |
| 175 | ACUTE\_MYELOID\_LEUKEMIA(HSA05221) |  | 96 | 0.58 | 1.04 | 0.000 | 0.415 | 1.000 | 21520 | tags=65%, list=36%, signal=101% |
| 176 | PATHWAYS\_IN\_CANCER(HSA05200) |  | 906 | 0.58 | 1.04 | 0.170 | 0.414 | 1.000 | 20534 | tags=47%, list=35%, signal=70% |
| 177 | SELENOCOMPOUND\_METABOLISM(HSA00450) |  | 28 | 0.60 | 1.04 | 0.170 | 0.413 | 1.000 | 22477 | tags=68%, list=38%, signal=109% |
| 178 | YERSINIA\_INFECTION(HSA05135) |  | 321 | 0.58 | 1.04 | 0.170 | 0.415 | 1.000 | 23009 | tags=45%, list=39%, signal=74% |
| 179 | PROTEIN\_EXPORT(HSA03060) |  | 57 | 0.62 | 1.04 | 0.165 | 0.414 | 1.000 | 19586 | tags=60%, list=33%, signal=89% |
| 180 | ENDOCYTOSIS(HSA04144) |  | 530 | 0.58 | 1.04 | 0.170 | 0.416 | 1.000 | 22688 | tags=57%, list=38%, signal=92% |
| 181 | DOPAMINERGIC\_SYNAPSE(HSA04728) |  | 185 | 0.58 | 1.04 | 0.000 | 0.415 | 1.000 | 19811 | tags=54%, list=34%, signal=80% |
| 182 | INSULIN\_SIGNALING\_PATHWAY(HSA04910) |  | 250 | 0.58 | 1.04 | 0.170 | 0.415 | 1.000 | 23875 | tags=63%, list=40%, signal=106% |
| 183 | TRYPTOPHAN\_METABOLISM(HSA00380) |  | 76 | 0.58 | 1.03 | 0.000 | 0.415 | 1.000 | 22984 | tags=61%, list=39%, signal=99% |
| 184 | RAS\_SIGNALING\_PATHWAY(HSA04014) |  | 413 | 0.58 | 1.03 | 0.000 | 0.418 | 1.000 | 22097 | tags=54%, list=37%, signal=86% |
| 185 | SPINOCEREBELLAR\_ATAXIA(HSA05017) |  | 399 | 0.57 | 1.03 | 0.170 | 0.417 | 1.000 | 24476 | tags=55%, list=41%, signal=94% |
| 186 | OXYTOCIN\_SIGNALING\_PATHWAY(HSA04921) |  | 261 | 0.58 | 1.03 | 0.170 | 0.422 | 1.000 | 19826 | tags=49%, list=34%, signal=74% |
| 187 | LONG\_TERM\_POTENTIATION(HSA04720) |  | 105 | 0.59 | 1.03 | 0.000 | 0.421 | 1.000 | 16882 | tags=48%, list=29%, signal=67% |
| 188 | TIGHT\_JUNCTION(HSA04530) |  | 324 | 0.58 | 1.03 | 0.170 | 0.421 | 1.000 | 23815 | tags=56%, list=40%, signal=94% |
| 189 | TUBERCULOSIS(HSA05152) |  | 389 | 0.57 | 1.03 | 0.000 | 0.423 | 1.000 | 20357 | tags=36%, list=34%, signal=54% |
| 190 | COLORECTAL\_CANCER(HSA05210) |  | 177 | 0.58 | 1.03 | 0.000 | 0.421 | 1.000 | 22789 | tags=55%, list=39%, signal=89% |
| 191 | INFLUENZA\_A(HSA05164) |  | 374 | 0.57 | 1.03 | 0.170 | 0.424 | 1.000 | 21106 | tags=41%, list=36%, signal=64% |
| 192 | FATTY\_ACID\_METABOLISM(HSA01212) |  | 84 | 0.59 | 1.03 | 0.000 | 0.423 | 1.000 | 22877 | tags=74%, list=39%, signal=120% |
| 193 | LYSOSOME(HSA04142) |  | 207 | 0.59 | 1.03 | 0.000 | 0.422 | 1.000 | 23266 | tags=74%, list=39%, signal=121% |
| 194 | GNRH\_SIGNALING\_PATHWAY(HSA04912) |  | 140 | 0.59 | 1.03 | 0.000 | 0.423 | 1.000 | 16884 | tags=49%, list=29%, signal=69% |
| 195 | GLYCOSAMINOGLYCAN\_BIOSYNTHESIS\_\_\_HEPARAN\_SULFATE\_\_\_HEPARIN(HSA00534) |  | 30 | 0.60 | 1.03 | 0.324 | 0.431 | 1.000 | 15944 | tags=50%, list=27%, signal=68% |
| 196 | VALINE\_\_LEUCINE\_AND\_ISOLEUCINE\_DEGRADATION(HSA00280) |  | 72 | 0.59 | 1.03 | 0.000 | 0.433 | 1.000 | 22877 | tags=74%, list=39%, signal=120% |
| 197 | CHEMOKINE\_SIGNALING\_PATHWAY(HSA04062) |  | 267 | 0.57 | 1.03 | 0.000 | 0.432 | 1.000 | 21106 | tags=47%, list=36%, signal=73% |
| 198 | GLYCOSAMINOGLYCAN\_BIOSYNTHESIS\_\_\_CHONDROITIN\_SULFATE\_\_\_DERMATAN\_SULFATE(HSA00532) |  | 28 | 0.61 | 1.03 | 0.324 | 0.432 | 1.000 | 16765 | tags=50%, list=28%, signal=70% |
| 199 | AMPK\_SIGNALING\_PATHWAY(HSA04152) |  | 174 | 0.58 | 1.03 | 0.154 | 0.437 | 1.000 | 23875 | tags=72%, list=40%, signal=121% |
| 200 | HIF\_1\_SIGNALING\_PATHWAY(HSA04066) |  | 307 | 0.57 | 1.03 | 0.170 | 0.438 | 1.000 | 20326 | tags=37%, list=34%, signal=56% |
| 201 | TH1\_AND\_TH2\_CELL\_DIFFERENTIATION(HSA04658) |  | 115 | 0.59 | 1.03 | 0.170 | 0.438 | 1.000 | 20351 | tags=55%, list=34%, signal=83% |
| 202 | ADIPOCYTOKINE\_SIGNALING\_PATHWAY(HSA04920) |  | 94 | 0.58 | 1.03 | 0.324 | 0.442 | 1.000 | 23256 | tags=67%, list=39%, signal=110% |
| 203 | PANCREATIC\_CANCER(HSA05212) |  | 135 | 0.58 | 1.03 | 0.000 | 0.444 | 1.000 | 22789 | tags=61%, list=39%, signal=100% |
| 204 | INOSITOL\_PHOSPHATE\_METABOLISM(HSA00562) |  | 107 | 0.58 | 1.03 | 0.000 | 0.442 | 1.000 | 23075 | tags=71%, list=39%, signal=116% |
| 205 | AXON\_GUIDANCE(HSA04360) |  | 283 | 0.58 | 1.03 | 0.324 | 0.443 | 1.000 | 24017 | tags=66%, list=41%, signal=111% |
| 206 | PROSTATE\_CANCER(HSA05215) |  | 157 | 0.58 | 1.03 | 0.000 | 0.444 | 1.000 | 23862 | tags=68%, list=40%, signal=114% |
| 207 | COMPLEMENT\_AND\_COAGULATION\_CASCADES(HSA04610) |  | 120 | 0.59 | 1.03 | 0.333 | 0.442 | 1.000 | 13035 | tags=29%, list=22%, signal=37% |
| 208 | LEUKOCYTE\_TRANSENDOTHELIAL\_MIGRATION(HSA04670) |  | 248 | 0.57 | 1.03 | 0.324 | 0.452 | 1.000 | 24242 | tags=54%, list=41%, signal=91% |
| 209 | TOXOPLASMOSIS(HSA05145) |  | 230 | 0.57 | 1.03 | 0.324 | 0.452 | 1.000 | 21414 | tags=40%, list=36%, signal=63% |
| 210 | PI3K\_AKT\_SIGNALING\_PATHWAY(HSA04151) |  | 654 | 0.56 | 1.03 | 0.170 | 0.457 | 1.000 | 19568 | tags=41%, list=33%, signal=60% |
| 211 | T\_CELL\_RECEPTOR\_SIGNALING\_PATHWAY(HSA04660) |  | 140 | 0.58 | 1.03 | 0.170 | 0.459 | 1.000 | 22747 | tags=59%, list=38%, signal=96% |
| 212 | HEPATITIS\_B(HSA05161) |  | 286 | 0.57 | 1.03 | 0.000 | 0.459 | 1.000 | 22868 | tags=54%, list=39%, signal=87% |
| 213 | GLUCAGON\_SIGNALING\_PATHWAY(HSA04922) |  | 185 | 0.58 | 1.03 | 0.324 | 0.459 | 1.000 | 23790 | tags=57%, list=40%, signal=96% |
| 214 | AMYOTROPHIC\_LATERAL\_SCLEROSIS\_(ALS)(HSA05014) |  | 135 | 0.57 | 1.02 | 0.154 | 0.462 | 1.000 | 17672 | tags=35%, list=30%, signal=50% |
| 215 | EGFR\_TYROSINE\_KINASE\_INHIBITOR\_RESISTANCE(HSA01521) |  | 166 | 0.57 | 1.02 | 0.333 | 0.463 | 1.000 | 19512 | tags=45%, list=33%, signal=67% |
| 216 | TYPE\_II\_DIABETES\_MELLITUS(HSA04930) |  | 68 | 0.58 | 1.02 | 0.324 | 0.464 | 1.000 | 23256 | tags=65%, list=39%, signal=107% |
| 217 | SMALL\_CELL\_LUNG\_CANCER(HSA05222) |  | 197 | 0.57 | 1.02 | 0.333 | 0.464 | 1.000 | 19286 | tags=39%, list=33%, signal=58% |
| 218 | GROWTH\_HORMONE\_SYNTHESIS\_\_SECRETION\_AND\_ACTION(HSA04935) |  | 165 | 0.58 | 1.02 | 0.170 | 0.465 | 1.000 | 23073 | tags=66%, list=39%, signal=108% |
| 219 | NATURAL\_KILLER\_CELL\_MEDIATED\_CYTOTOXICITY(HSA04650) |  | 281 | 0.57 | 1.02 | 0.324 | 0.471 | 1.000 | 21590 | tags=34%, list=37%, signal=53% |
| 220 | FC\_EPSILON\_RI\_SIGNALING\_PATHWAY(HSA04664) |  | 153 | 0.57 | 1.02 | 0.000 | 0.472 | 1.000 | 23896 | tags=42%, list=40%, signal=70% |
| 221 | SYNAPTIC\_VESICLE\_CYCLE(HSA04721) |  | 138 | 0.58 | 1.02 | 0.324 | 0.472 | 1.000 | 21311 | tags=51%, list=36%, signal=80% |
| 222 | JAK\_STAT\_SIGNALING\_PATHWAY(HSA04630) |  | 236 | 0.57 | 1.02 | 0.333 | 0.476 | 1.000 | 23872 | tags=56%, list=40%, signal=93% |
| 223 | MAPK\_SIGNALING\_PATHWAY(HSA04010) |  | 485 | 0.57 | 1.02 | 0.333 | 0.478 | 1.000 | 22857 | tags=57%, list=39%, signal=92% |
| 224 | PHOSPHOLIPASE\_D\_SIGNALING\_PATHWAY(HSA04072) |  | 295 | 0.57 | 1.02 | 0.324 | 0.478 | 1.000 | 22747 | tags=47%, list=38%, signal=77% |
| 225 | FLUID\_SHEAR\_STRESS\_AND\_ATHEROSCLEROSIS(HSA05418) |  | 328 | 0.57 | 1.02 | 0.333 | 0.479 | 1.000 | 23308 | tags=48%, list=39%, signal=79% |
| 226 | SPHINGOLIPID\_METABOLISM(HSA00600) |  | 72 | 0.58 | 1.02 | 0.154 | 0.477 | 1.000 | 21751 | tags=60%, list=37%, signal=94% |
| 227 | PROTEOGLYCANS\_IN\_CANCER(HSA05205) |  | 384 | 0.57 | 1.02 | 0.324 | 0.476 | 1.000 | 23896 | tags=58%, list=40%, signal=96% |
| 228 | MITOPHAGY\_\_\_ANIMAL(HSA04137) |  | 330 | 0.56 | 1.02 | 0.000 | 0.477 | 1.000 | 24042 | tags=47%, list=41%, signal=79% |
| 229 | OTHER\_GLYCAN\_DEGRADATION(HSA00511) |  | 27 | 0.61 | 1.02 | 0.154 | 0.479 | 1.000 | 22805 | tags=78%, list=39%, signal=127% |
| 230 | ADRENERGIC\_SIGNALING\_IN\_CARDIOMYOCYTES(HSA04261) |  | 207 | 0.57 | 1.02 | 0.000 | 0.480 | 1.000 | 21224 | tags=55%, list=36%, signal=86% |
| 231 | HEPATITIS\_C(HSA05160) |  | 292 | 0.57 | 1.02 | 0.000 | 0.480 | 1.000 | 24723 | tags=55%, list=42%, signal=94% |
| 232 | C\_TYPE\_LECTIN\_RECEPTOR\_SIGNALING\_PATHWAY(HSA04625) |  | 158 | 0.57 | 1.02 | 0.333 | 0.478 | 1.000 | 23306 | tags=62%, list=39%, signal=102% |
| 233 | ALANINE\_\_ASPARTATE\_AND\_GLUTAMATE\_METABOLISM(HSA00250) |  | 96 | 0.57 | 1.02 | 0.000 | 0.477 | 1.000 | 18124 | tags=40%, list=31%, signal=57% |
| 234 | HUMAN\_PAPILLOMAVIRUS\_INFECTION(HSA05165) |  | 544 | 0.57 | 1.02 | 0.324 | 0.478 | 1.000 | 24291 | tags=63%, list=41%, signal=105% |
| 235 | SEROTONERGIC\_SYNAPSE(HSA04726) |  | 164 | 0.57 | 1.02 | 0.154 | 0.478 | 1.000 | 15945 | tags=37%, list=27%, signal=50% |
| 236 | CGMP\_PKG\_SIGNALING\_PATHWAY(HSA04022) |  | 309 | 0.57 | 1.02 | 0.333 | 0.482 | 1.000 | 18447 | tags=44%, list=31%, signal=64% |
| 237 | DILATED\_CARDIOMYOPATHY\_(DCM)(HSA05414) |  | 245 | 0.58 | 1.02 | 0.487 | 0.484 | 1.000 | 12604 | tags=24%, list=21%, signal=30% |
| 238 | OTHER\_TYPES\_OF\_O\_GLYCAN\_BIOSYNTHESIS(HSA00514) |  | 67 | 0.58 | 1.02 | 0.000 | 0.486 | 1.000 | 22394 | tags=63%, list=38%, signal=101% |
| 239 | CORTISOL\_SYNTHESIS\_AND\_SECRETION(HSA04927) |  | 94 | 0.59 | 1.02 | 0.317 | 0.486 | 1.000 | 16065 | tags=38%, list=27%, signal=52% |
| 240 | TRANSCRIPTIONAL\_MISREGULATION\_IN\_CANCER(HSA05202) |  | 381 | 0.57 | 1.02 | 0.324 | 0.491 | 1.000 | 20726 | tags=42%, list=35%, signal=64% |
| 241 | INSULIN\_RESISTANCE(HSA04931) |  | 143 | 0.58 | 1.02 | 0.170 | 0.494 | 1.000 | 23875 | tags=75%, list=40%, signal=125% |
| 242 | TOLL\_LIKE\_RECEPTOR\_SIGNALING\_PATHWAY(HSA04620) |  | 135 | 0.58 | 1.02 | 0.163 | 0.498 | 1.000 | 22868 | tags=57%, list=39%, signal=93% |
| 243 | PHOSPHATIDYLINOSITOL\_SIGNALING\_SYSTEM(HSA04070) |  | 141 | 0.58 | 1.02 | 0.163 | 0.501 | 1.000 | 24476 | tags=76%, list=41%, signal=129% |
| 244 | APELIN\_SIGNALING\_PATHWAY(HSA04371) |  | 242 | 0.56 | 1.02 | 0.333 | 0.502 | 1.000 | 20945 | tags=47%, list=35%, signal=73% |
| 245 | CAMP\_SIGNALING\_PATHWAY(HSA04024) |  | 309 | 0.57 | 1.01 | 0.333 | 0.502 | 1.000 | 23645 | tags=57%, list=40%, signal=94% |
| 246 | ARRHYTHMOGENIC\_RIGHT\_VENTRICULAR\_CARDIOMYOPATHY\_(ARVC)(HSA05412) |  | 174 | 0.57 | 1.01 | 0.333 | 0.501 | 1.000 | 21162 | tags=45%, list=36%, signal=70% |
| 247 | FOXO\_SIGNALING\_PATHWAY(HSA04068) |  | 244 | 0.57 | 1.01 | 0.183 | 0.502 | 1.000 | 24837 | tags=64%, list=42%, signal=109% |
| 248 | TGF\_BETA\_SIGNALING\_PATHWAY(HSA04350) |  | 148 | 0.58 | 1.01 | 0.324 | 0.500 | 1.000 | 22211 | tags=59%, list=38%, signal=95% |
| 249 | MEASLES(HSA05162) |  | 267 | 0.56 | 1.01 | 0.333 | 0.500 | 1.000 | 23372 | tags=47%, list=40%, signal=77% |
| 250 | FERROPTOSIS(HSA04216) |  | 124 | 0.57 | 1.01 | 0.333 | 0.499 | 1.000 | 23363 | tags=45%, list=40%, signal=74% |
| 251 | STARCH\_AND\_SUCROSE\_METABOLISM(HSA00500) |  | 51 | 0.57 | 1.01 | 0.324 | 0.498 | 1.000 | 22743 | tags=49%, list=38%, signal=80% |
| 252 | FATTY\_ACID\_DEGRADATION(HSA00071) |  | 74 | 0.57 | 1.01 | 0.165 | 0.506 | 1.000 | 22877 | tags=58%, list=39%, signal=95% |
| 253 | ERBB\_SIGNALING\_PATHWAY(HSA04012) |  | 121 | 0.58 | 1.01 | 0.170 | 0.507 | 1.000 | 23896 | tags=69%, list=40%, signal=116% |
| 254 | RENIN\_SECRETION(HSA04924) |  | 101 | 0.57 | 1.01 | 0.333 | 0.514 | 1.000 | 17677 | tags=45%, list=30%, signal=63% |
| 255 | SNARE\_INTERACTIONS\_IN\_VESICULAR\_TRANSPORT(HSA04130) |  | 57 | 0.59 | 1.01 | 0.328 | 0.521 | 1.000 | 23252 | tags=68%, list=39%, signal=113% |
| 256 | ALDOSTERONE\_SYNTHESIS\_AND\_SECRETION(HSA04925) |  | 143 | 0.57 | 1.01 | 0.487 | 0.524 | 1.000 | 18248 | tags=45%, list=31%, signal=66% |
| 257 | RIG\_I\_LIKE\_RECEPTOR\_SIGNALING\_PATHWAY(HSA04622) |  | 101 | 0.58 | 1.01 | 0.487 | 0.528 | 1.000 | 20026 | tags=50%, list=34%, signal=75% |
| 258 | AMPHETAMINE\_ADDICTION(HSA05031) |  | 94 | 0.57 | 1.01 | 0.487 | 0.529 | 1.000 | 24199 | tags=65%, list=41%, signal=110% |
| 259 | PPAR\_SIGNALING\_PATHWAY(HSA03320) |  | 135 | 0.56 | 1.01 | 0.333 | 0.529 | 1.000 | 24087 | tags=53%, list=41%, signal=89% |
| 260 | ADHERENS\_JUNCTION(HSA04520) |  | 162 | 0.57 | 1.01 | 0.333 | 0.532 | 1.000 | 23330 | tags=56%, list=39%, signal=92% |
| 261 | HEMATOPOIETIC\_CELL\_LINEAGE(HSA04640) |  | 191 | 0.55 | 1.01 | 0.333 | 0.536 | 1.000 | 24861 | tags=31%, list=42%, signal=54% |
| 262 | OSTEOCLAST\_DIFFERENTIATION(HSA04380) |  | 172 | 0.57 | 1.01 | 0.487 | 0.535 | 1.000 | 23256 | tags=58%, list=39%, signal=95% |
| 263 | CHOLINE\_METABOLISM\_IN\_CANCER(HSA05231) |  | 151 | 0.57 | 1.00 | 0.487 | 0.548 | 1.000 | 24164 | tags=68%, list=41%, signal=115% |
| 264 | VITAMIN\_DIGESTION\_AND\_ABSORPTION(HSA04977) |  | 41 | 0.58 | 1.00 | 0.324 | 0.548 | 1.000 | 19381 | tags=41%, list=33%, signal=62% |
| 265 | AUTOPHAGY\_\_\_ANIMAL(HSA04140) |  | 448 | 0.56 | 1.00 | 0.346 | 0.550 | 1.000 | 24360 | tags=53%, list=41%, signal=90% |
| 266 | BILE\_SECRETION(HSA04976) |  | 162 | 0.56 | 1.00 | 0.324 | 0.552 | 1.000 | 15733 | tags=28%, list=27%, signal=39% |
| 267 | COCAINE\_ADDICTION(HSA05030) |  | 65 | 0.58 | 1.00 | 0.324 | 0.558 | 1.000 | 23578 | tags=65%, list=40%, signal=107% |
| 268 | FOCAL\_ADHESION(HSA04510) |  | 377 | 0.56 | 1.00 | 0.487 | 0.568 | 1.000 | 24608 | tags=58%, list=42%, signal=98% |
| 269 | NOD\_LIKE\_RECEPTOR\_SIGNALING\_PATHWAY(HSA04621) |  | 345 | 0.56 | 1.00 | 0.487 | 0.571 | 1.000 | 24579 | tags=50%, list=42%, signal=85% |
| 270 | TH17\_CELL\_DIFFERENTIATION(HSA04659) |  | 147 | 0.57 | 1.00 | 0.487 | 0.572 | 1.000 | 20702 | tags=47%, list=35%, signal=72% |
| 271 | AUTOPHAGY\_\_\_OTHER(HSA04136) |  | 58 | 0.57 | 1.00 | 0.317 | 0.570 | 1.000 | 24176 | tags=69%, list=41%, signal=117% |
| 272 | COLLECTING\_DUCT\_ACID\_SECRETION(HSA04966) |  | 52 | 0.58 | 1.00 | 0.487 | 0.569 | 1.000 | 24460 | tags=60%, list=41%, signal=102% |
| 273 | IL\_17\_SIGNALING\_PATHWAY(HSA04657) |  | 142 | 0.57 | 1.00 | 0.487 | 0.585 | 1.000 | 19286 | tags=46%, list=33%, signal=68% |
| 274 | RETINOL\_METABOLISM(HSA00830) |  | 148 | 0.57 | 1.00 | 0.487 | 0.586 | 1.000 | 16370 | tags=26%, list=28%, signal=35% |
| 275 | CYTOKINE\_CYTOKINE\_RECEPTOR\_INTERACTION(HSA04060) |  | 353 | 0.56 | 1.00 | 0.487 | 0.588 | 1.000 | 24214 | tags=49%, list=41%, signal=82% |
| 276 | EPITHELIAL\_CELL\_SIGNALING\_IN\_HELICOBACTER\_PYLORI\_INFECTION(HSA05120) |  | 128 | 0.56 | 1.00 | 0.487 | 0.594 | 1.000 | 24583 | tags=66%, list=42%, signal=112% |
| 277 | LONGEVITY\_REGULATING\_PATHWAY(HSA04211) |  | 136 | 0.56 | 1.00 | 0.333 | 0.595 | 1.000 | 24331 | tags=64%, list=41%, signal=108% |
| 278 | PANCREATIC\_SECRETION(HSA04972) |  | 159 | 0.56 | 0.99 | 0.487 | 0.606 | 1.000 | 21187 | tags=41%, list=36%, signal=64% |
| 279 | CARDIAC\_MUSCLE\_CONTRACTION(HSA04260) |  | 377 | 0.54 | 0.99 | 0.333 | 0.607 | 1.000 | 24233 | tags=26%, list=41%, signal=44% |
| 280 | GLYCOSPHINGOLIPID\_BIOSYNTHESIS\_\_\_GANGLIO\_SERIES(HSA00604) |  | 21 | 0.59 | 0.99 | 0.500 | 0.608 | 1.000 | 22930 | tags=71%, list=39%, signal=117% |
| 281 | AMOEBIASIS(HSA05146) |  | 210 | 0.55 | 0.99 | 0.487 | 0.611 | 1.000 | 22097 | tags=33%, list=37%, signal=52% |
| 282 | PLATINUM\_DRUG\_RESISTANCE(HSA01524) |  | 159 | 0.57 | 0.99 | 0.482 | 0.612 | 1.000 | 24024 | tags=50%, list=41%, signal=83% |
| 283 | REGULATION\_OF\_LIPOLYSIS\_IN\_ADIPOCYTES(HSA04923) |  | 78 | 0.57 | 0.99 | 0.487 | 0.613 | 1.000 | 24838 | tags=62%, list=42%, signal=106% |
| 284 | PRIMARY\_BILE\_ACID\_BIOSYNTHESIS(HSA00120) |  | 27 | 0.57 | 0.99 | 0.516 | 0.619 | 1.000 | 22908 | tags=56%, list=39%, signal=91% |
| 285 | GLYCEROLIPID\_METABOLISM(HSA00561) |  | 103 | 0.57 | 0.99 | 0.487 | 0.618 | 1.000 | 24892 | tags=59%, list=42%, signal=102% |
| 286 | CHAGAS\_DISEASE\_(AMERICAN\_TRYPANOSOMIASIS)(HSA05142) |  | 144 | 0.57 | 0.99 | 0.333 | 0.619 | 1.000 | 22747 | tags=52%, list=38%, signal=84% |
| 287 | PERTUSSIS(HSA05133) |  | 125 | 0.56 | 0.99 | 0.487 | 0.625 | 1.000 | 23256 | tags=47%, list=39%, signal=78% |
| 288 | OLFACTORY\_TRANSDUCTION(HSA04740) |  | 950 | 0.49 | 0.99 | 0.333 | 0.624 | 1.000 | 24371 | tags=11%, list=41%, signal=18% |
| 289 | APOPTOSIS\_\_\_MULTIPLE\_SPECIES(HSA04215) |  | 88 | 0.56 | 0.99 | 0.487 | 0.623 | 1.000 | 23932 | tags=48%, list=40%, signal=80% |
| 290 | HERPES\_SIMPLEX\_VIRUS\_1\_INFECTION(HSA05168) |  | 1043 | 0.56 | 0.99 | 0.511 | 0.623 | 1.000 | 25241 | tags=55%, list=43%, signal=94% |
| 291 | GLYCEROPHOSPHOLIPID\_METABOLISM(HSA00564) |  | 143 | 0.56 | 0.99 | 0.487 | 0.624 | 1.000 | 24953 | tags=69%, list=42%, signal=119% |
| 292 | NF\_KAPPA\_B\_SIGNALING\_PATHWAY(HSA04064) |  | 201 | 0.56 | 0.99 | 0.487 | 0.636 | 1.000 | 24342 | tags=43%, list=41%, signal=73% |
| 293 | GLYCOSAMINOGLYCAN\_BIOSYNTHESIS\_\_\_KERATAN\_SULFATE(HSA00533) |  | 18 | 0.60 | 0.98 | 0.324 | 0.649 | 1.000 | 22201 | tags=78%, list=38%, signal=124% |
| 294 | PROTEIN\_DIGESTION\_AND\_ABSORPTION(HSA04974) |  | 128 | 0.56 | 0.98 | 0.487 | 0.652 | 1.000 | 17332 | tags=35%, list=29%, signal=50% |
| 295 | STEROID\_HORMONE\_BIOSYNTHESIS(HSA00140) |  | 136 | 0.54 | 0.98 | 0.670 | 0.658 | 1.000 | 23382 | tags=32%, list=40%, signal=52% |
| 296 | TNF\_SIGNALING\_PATHWAY(HSA04668) |  | 173 | 0.56 | 0.98 | 0.835 | 0.688 | 1.000 | 24342 | tags=62%, list=41%, signal=106% |
| 297 | HIPPO\_SIGNALING\_PATHWAY\_\_\_MULTIPLE\_SPECIES(HSA04392) |  | 40 | 0.58 | 0.97 | 0.482 | 0.698 | 1.000 | 23944 | tags=68%, list=40%, signal=113% |
| 298 | AGE\_RAGE\_SIGNALING\_PATHWAY\_IN\_DIABETIC\_COMPLICATIONS(HSA04933) |  | 134 | 0.56 | 0.97 | 0.835 | 0.707 | 1.000 | 22887 | tags=56%, list=39%, signal=91% |
| 299 | VIRAL\_PROTEIN\_INTERACTION\_WITH\_CYTOKINE\_AND\_CYTOKINE\_RECEPTOR(HSA04061) |  | 114 | 0.58 | 0.97 | 0.317 | 0.713 | 1.000 | 12572 | tags=24%, list=21%, signal=30% |
| 300 | AUTOIMMUNE\_THYROID\_DISEASE(HSA05320) |  | 159 | 0.56 | 0.97 | 0.487 | 0.712 | 1.000 | 10733 | tags=8%, list=18%, signal=10% |
| 301 | NICOTINATE\_AND\_NICOTINAMIDE\_METABOLISM(HSA00760) |  | 58 | 0.55 | 0.97 | 0.487 | 0.716 | 1.000 | 25684 | tags=64%, list=43%, signal=113% |
| 302 | CELL\_ADHESION\_MOLECULES\_(CAMS)(HSA04514) |  | 216 | 0.56 | 0.97 | 0.487 | 0.718 | 1.000 | 24438 | tags=47%, list=41%, signal=79% |
| 303 | BETA\_ALANINE\_METABOLISM(HSA00410) |  | 47 | 0.56 | 0.97 | 0.652 | 0.732 | 1.000 | 24654 | tags=64%, list=42%, signal=109% |
| 304 | ETHER\_LIPID\_METABOLISM(HSA00565) |  | 72 | 0.56 | 0.96 | 0.670 | 0.763 | 1.000 | 23032 | tags=54%, list=39%, signal=89% |
| 305 | HYPERTROPHIC\_CARDIOMYOPATHY\_(HCM)(HSA05410) |  | 174 | 0.54 | 0.96 | 0.487 | 0.808 | 1.000 | 24733 | tags=51%, list=42%, signal=87% |
| 306 | CIRCADIAN\_RHYTHM(HSA04710) |  | 53 | 0.55 | 0.96 | 0.835 | 0.809 | 1.000 | 26021 | tags=64%, list=44%, signal=114% |
| 307 | ECM\_RECEPTOR\_INTERACTION(HSA04512) |  | 115 | 0.55 | 0.95 | 0.487 | 0.826 | 1.000 | 26398 | tags=74%, list=45%, signal=133% |
| 308 | ALLOGRAFT\_REJECTION(HSA05330) |  | 142 | 0.56 | 0.95 | 0.487 | 0.841 | 1.000 | 20131 | tags=13%, list=34%, signal=19% |
| 309 | MUCIN\_TYPE\_O\_GLYCAN\_BIOSYNTHESIS(HSA00512) |  | 49 | 0.55 | 0.95 | 0.835 | 0.849 | 1.000 | 26231 | tags=65%, list=44%, signal=117% |
| 310 | RHEUMATOID\_ARTHRITIS(HSA05323) |  | 198 | 0.54 | 0.95 | 0.670 | 0.856 | 1.000 | 22979 | tags=29%, list=39%, signal=47% |
| 311 | RIBOFLAVIN\_METABOLISM(HSA00740) |  | 19 | 0.59 | 0.95 | 0.507 | 0.865 | 1.000 | 20291 | tags=42%, list=34%, signal=64% |
| 312 | CHEMICAL\_CARCINOGENESIS(HSA05204) |  | 180 | 0.53 | 0.95 | 0.835 | 0.866 | 1.000 | 24966 | tags=33%, list=42%, signal=57% |
| 313 | METABOLISM\_OF\_XENOBIOTICS\_BY\_CYTOCHROME\_P450(HSA00980) |  | 168 | 0.53 | 0.95 | 0.652 | 0.865 | 1.000 | 25973 | tags=35%, list=44%, signal=62% |
| 314 | FAT\_DIGESTION\_AND\_ABSORPTION(HSA04975) |  | 73 | 0.55 | 0.94 | 0.670 | 0.880 | 1.000 | 24659 | tags=45%, list=42%, signal=77% |
| 315 | MALARIA(HSA05144) |  | 62 | 0.54 | 0.94 | 0.835 | 0.905 | 1.000 | 26572 | tags=56%, list=45%, signal=102% |
| 316 | TYPE\_I\_DIABETES\_MELLITUS(HSA04940) |  | 106 | 0.54 | 0.94 | 0.487 | 0.921 | 1.000 | 24215 | tags=28%, list=41%, signal=48% |
| 317 | INFLAMMATORY\_BOWEL\_DISEASE\_(IBD)(HSA05321) |  | 79 | 0.54 | 0.92 | 0.835 | 0.958 | 1.000 | 26572 | tags=54%, list=45%, signal=99% |
| 318 | HISTIDINE\_METABOLISM(HSA00340) |  | 31 | 0.55 | 0.92 | 0.652 | 0.969 | 1.000 | 25273 | tags=71%, list=43%, signal=124% |
| 319 | PANTOTHENATE\_AND\_COA\_BIOSYNTHESIS(HSA00770) |  | 24 | 0.56 | 0.92 | 0.835 | 0.968 | 1.000 | 26145 | tags=75%, list=44%, signal=134% |
| 320 | GRAFT\_VERSUS\_HOST\_DISEASE(HSA05332) |  | 80 | 0.55 | 0.91 | 0.487 | 0.973 | 1.000 | 20131 | tags=18%, list=34%, signal=26% |
| 321 | ARACHIDONIC\_ACID\_METABOLISM(HSA00590) |  | 126 | 0.54 | 0.91 | 0.670 | 0.978 | 1.000 | 24548 | tags=44%, list=42%, signal=74% |
| 322 | DRUG\_METABOLISM\_\_\_CYTOCHROME\_P450(HSA00982) |  | 167 | 0.52 | 0.90 | 0.835 | 0.980 | 1.000 | 26426 | tags=33%, list=45%, signal=59% |
| 323 | LINOLEIC\_ACID\_METABOLISM(HSA00591) |  | 55 | 0.54 | 0.89 | 0.670 | 0.984 | 1.000 | 24953 | tags=45%, list=42%, signal=79% |
| 324 | ALPHA\_LINOLENIC\_ACID\_METABOLISM(HSA00592) |  | 40 | 0.54 | 0.89 | 0.670 | 0.985 | 1.000 | 24953 | tags=55%, list=42%, signal=95% |
Table: Gene sets enriched in phenotype
 **WT\_VIM (2 samples)
 **[plain text format]****
